# The human RNASET2 alarmin-like molecule differentially affects prostate cancer cells behavior in both cell autonomous and non-cell autonomous manners

**DOI:** 10.1186/s12967-025-06540-0

**Published:** 2025-05-19

**Authors:** Rossella Roncoroni, Denisa Baci, Martina Cucchiara, Annarosaria de Vito, Matteo Gallazzi, Maria Teresa Palano, Francesca Olmeo, Cristian Rubuano, Alessandra Giannatiempo, Laura Monti, Raffaella Bombelli, Daniele Mercatelli, Giovanna Finzi, Francesca Franzi, Stefano La Rosa, Douglas M. Noonan, Barbara Bassani, Lorenzo Mortara, Antonino Bruno, Francesco Acquati

**Affiliations:** 1 Laboratory of Human Genetics, Department of Biotechnology and Life Sciences, University of Insubria, Varese, Italy; 2 Laboratory of Immunology and General Pathology, Department of Biotechnology and Life Sciences, University of Insubria, Varese, Italy; 3 Molecular Cardiology Laboratory, IRCCS-Policlinico San Donato, San Donato Milanese, Milan, Italy; 4 Laboratory of Innate Immunity, Unit of Molecular Pathology, Biochemistry, and Immunology, IRCCS MultiMedica, Milan, Italy; 5 Department of Pharmacy and Biotechnology, University of Bologna, Bologna, Italy; 6 Unit of Pathology, Department of Oncology, ASST Sette Laghi, Varese, Italy; 7 Unit of Pathology, Department of Medicine and Technological Innovation, University of Insubria, Varese, Italy; 8 Hereditary Cancer Research Center, University of Insubria, Varese, Italy; 9 Unit of Molecular Pathology, Biochemistry, and Immunology IRCCS MultiMedica, Milan, Italy

**Keywords:** Alarmins, RNASET2, Prostate cancer, Oncosuppressors, Macrophages

## Abstract

The identification of molecules that make cancer cells detectable by the immune system represents a major challenge in tumor immunology. Alarmins, endogenous, stress-induced molecules, serve as early warning signals triggering immune responses. The human RNASET2 protein has demonstrated both oncosuppressive and immunoregulatory functions across various cancer types, yet its role as an oncosuppressor or alarmin-like molecule in prostate cancer (PCa) is unexplored. Here, we investigated the effects of the human RNASET2 alarmin in two different human PCa cell lines focusing on cell proliferation, colony formation, adhesion, migration rates, and release of soluble immune-modulatory factors. In vivo studies were also carried out on nude mice to assess the immune regulatory impact. Our findings indicate that RNASET2 overexpression reduced cell proliferation and colony formation in 22Rv1 cells, through downregulation of cyclin D1. RNASET2 overexpression in 22Rv1 cells was also associated with decreased levels of *TWIST*,* CTNNB1*,* YAP*,* and MMP-9*. By contrast, PC-3 cells were largely unresponsive to RNASET2. RNASET2 overexpression also promoted the release of soluble factors related to monocyte/macrophage recruitment/activation and cytokines/chemokines, linked to immune cell-mediated anti-tumor responses. This effect was more pronounced in RNASET2-overexpressing 22Rv1 cells and involved both innate (NK cells, dendritic cells) and adaptive (T cells) immune activation, compared to PC-3 cells. RNASET2 overexpression also affected the cytoskeletal organization in both PCa models. RNASET2 overexpression in vivo induced a shift toward M1-like macrophage polarization pattern, while decreasing the M2-like polarization in mice challenged with 22Rv1 cells, indicating a potential tumor-suppressive role in PCa. Finally, silencing of RNASET2 in THP-1 macrophages unveiled their phagocytic activities against PCa cells. Our findings underscore the RNASET2’s dual functionality, acting through both cell-autonomous and non-cell autonomous mechanisms in PCa i*n vitro* and in *vivo* models and suggest its potential as a therapeutic target in a subset of PCa.

**Supplementary Information:**

The online version contains supplementary material available at 10.1186/s12967-025-06540-0.

## Introduction

Prostate cancer (PCa) accounts for the most diagnosed neoplasm in men and ranks as the second leading cause of male cancer-related deaths worldwide [1]. Chronic inflammation is a recognized hallmark of PCa [2], contributing to both its onset [3] and progression toward metastasis [4–6].

RNASET2, a highly conserved extracellular protein belonging to the Rh/T2/s ribonuclease family [7], has been consistently reported as an oncosuppressor which can inhibit tumor growth in vivo, independently from its catalytic activity, with expression patterns shifting downward from early to late tumor stages [7]. The in vivo oncosuppressive activity of RNASET2 involves the establishment of a crosstalk between RNASET2-expressing cancer cells and components of the tumor microenvironment (TME) belonging to the innate immune system (mostly tissue macrophages) [7].

Recent data suggest that human *RNASET2* functions as a stress-response gene [8], potentially belonging to the “alarmin” family of extracellular immune effectors [9, 10]. Indeed, the observed RNASET2 expression changes from early (up-regulation) to late tumors (down-regulation), coupled to its role as a stress response gene [8, 10–12], are in keeping with this working hypothesis [13]. Accordingly, we discovered that the RNASET2 anticancer role is associated with recruitment [7] and polarization [7, 14] of myeloid cells endowed with anti-tumoral activity in both in vitro and in vivo experimental models. Immune cells themselves upregulate RNASET2 upon activation [11, 13].

Moreover, silencing of *RNASET2* in human macrophages down-regulates M1 markers, while upregulating M2 markers, supporting its tumor suppressive role within the TME [14]. Of note, the enzymatic activity of RNASET2 has also been reported to represent a non-redundant upstream component of TLR8-dependent activation of the innate immune system [15, 16].

We also demonstrated that overexpression of murine *Rnaset2* in a colon syngeneic mouse carcinoma model increased the intra-tumor M1/M2 macrophage ratio, activated T cells, delayed tumor growth, and led to tumor rejection, further confirming and extending the role of T2 RNases as immune-modulators in anti-tumor immune response [17].

Macrophages and monocytes, like other inflammatory cells, possess a high level of phenotype and functional plasticity that allows them to shift between M1-like or M2-like polarization states, based on the pathophysiological microenvironment [18, 19]. M1-like macrophages are typically associated with pro-inflammatory and antitumoral activities, whereas M2-like macrophages - or tumor-associated macrophages (TAMs) in the TME - exert pro-tumor functions, including immunosuppressive activities, pro-angiogenic and pro-metastatic activities [20, 21]. RNASET2 has also been involved in dendritic cell (DC) maturation, highlighting its potential role in innate immunity [13, 22, 23], and extracellular secreted RNASET2 can inhibit a well-established cancer-promoting process such as angiogenesis [24].

In PCa, both epithelial and stromal cells within the TME can release monocyte chemotactic protein (MCP)-1/C-C motif ligand (CCL2) along with other soluble factors, such as TGFβ, which fosters pro-tumor M2-like state polarization [25, 26] and correlates with tumor M2-like expansion and metastasis [27, 28]. Moreover, castration-resistant PCa patients with elevated absolute monocyte count show poor clinical outcomes, and the expansion of macrophages and T regulatory (T_reg_) cell populations within the TME has been reported as an adverse prognostic factor [29, 30].

In addition to the above-described non-cell autonomous role in tumor suppression, mediated primarily through the crosstalk with key components of the innate immune system, RNASET2 has also shown independent cell-autonomous tumor-suppressive mechanisms. Specifically, a wide range of cancer-related parameters, such as cell proliferation, clonogenic potential, anchorage-independent growth, and cell adhesion/invasion, have been reported to be negatively regulated by RNASET2 in several in vitro cellular models [8, 31–33]. Moreover, RNASET2 has been shown to induce both apoptosis and cellular senescence in independent cellular models [8, 12, 34].

A further potential cell-autonomous role for RNASET2 as an oncosuppressor protein has been recently reported, based on its ability to process the *TERC* RNA component of human telomerase complex within the mitochondria, thereby regulating cellular senescence independently of telomerase activity [34].

Thus, a solid body of evidence gathered over the last two decades points to *RNASET2* as a highly pleiotropic tumor suppressor gene, endowed with the ability to counteract cancer cell growth by several independent mechanisms. However, the role played by human *RNASET2* in the context of PCa has not been investigated to date.

Given these premises, we investigated whether RNASET2 overexpression might impact key features of PCa cells, such as cell proliferation, adhesion, and migration, using two independent cell lines. Considering a recent report pointing at RNASET2 as a candidate biomarker for low vs. high-grade PCa [40], we chose two cell lines (PC-3 and 22Rv1) representing poorly and highly aggressive forms of this tumor type, respectively, to investigate putative differences in their response to RNASET2 modulation.

We also evaluated the role of RNASET2 in modulating the ability of the abovementioned PCa cells to release immunomodulatory soluble factors involved in monocyte recruitment and pro-tumor immune cell polarization and activation. Finally, we validated our in vitro findings in an in vivo setting, using nude mice xenografted with RNASET2-expressing PC-3 and 22Rv1 PCa cells, as compared to their empty-vector transfected cells. Our findings were further supported by an extensive in silico analysis and strongly indicated that RNASET2’s established tumor-suppressive role extends to at least a subset of prostate cancer types.

## Materials and methods

### Cell line culture and maintenance

PC-3 and 22Rv1 human prostate cancer cell lines (purchased by ATCC) were maintained in RPMI 1640 medium, supplemented with 10% fetal bovine serum (FBS) (Euroclone), 2 mM L-glutamine (Euroclone), 100 U/ml penicillin and 100 µg/ml streptomycin (Euroclone), at 37 °C, 5% CO_2_. Cells were routinely screened by PCR for putative mycoplasma contaminations. For stably transfected pools and clones, the growth medium was supplemented with G418 (600 µg/mL for 22RV1 and 250 µg/mL for PC-3).

### Generation of RNASET2-overexpressing prostate cancer cell lines

RNASET2-overexpressing PC-3 and 22Rv1 prostate cancer cell lines were generated by stable transfection with a pcDNA3-based RNASET2 [33] expression vector using Lipofectamine (Invitrogen) according to the manufacturer’s instructions. As controls, cells were transfected with the empty pcDNA3 plasmid (empty vector). RNASET2 overexpression in both cell extracts and supernatants was confirmed and monitored by western blot analysis using a rabbit polyclonal anti-hRNASET2 antibody (Davids Biotechnologie GmbH, Regensburg, Germany).

For co-culture assays with THP-1-derived macrophages, PC-3 and 22Rv1 cells were first transfected with pcDNA3 expression vector coupled with the pEGFP-N1 vector encoding the green fluorescent protein (GFP). Pools of transfected cells were then FACS-sorted to obtain homogenous populations of GFP-overexpressing cells. After sorting, cells were either propagated as cellular pools or seeded by limiting dilution in 96-well plates to isolate single control clones.

### Generation of RNASET2-silenced THP-1 cells

The human promonocytic THP-1 cell line was cultured in RPMI-1640 media, with 10% heat-inactivated FBS and 1% glutamine. Human *RNASET2* knock-down THP-1 cells have been previously described [14]. To maintain *hRNASET2*-silenced THP-1 cells in culture, puromycin selection at a final concentration of 0,75 µg/ml was applied.

### Cell proliferation

The proliferation capacity of PC-3 or 22Rv1 PCa cell line, based on RNASET2 expression, was evaluated by the crystal violet colorimetric assay. A total of 4 × 10^3^ RNASET2-overexpressing and control PC-3 or 22Rv1 cells were seeded into 96-well plates and allowed to attach. Cell proliferation was assessed at 24, 48, and 78 h after attachment. Briefly, at each time point, cells were washed with PBS containing Ca^2+^/Mg^2+^ and stained with 50 µl of crystal violet staining solution (Sigma Aldrich) for 20 min at room temperature under gentle shaking. After three washes in distilled water, plates were left to dry for 2 h at room temperature. Finally, cell-retained crystal violet was dissolved in 100 µL of crystal violet elution buffer (50% ethanol and 0.1% acetic acid) and cell proliferation was determined by measuring absorbance at 595 nm using a SpectraMax M2 microplate reader (Molecular Devices, Sunnyvale CA).

For viability detection of *wt* or sh RNASET2 THP-1 cells, the XTT assay was used. A total of 3 × 10^4^ cells were plated into 96-well plates and monitored for viability at T0, 24, 48, and 72 h after seeding. At each time point, cells were incubated with 50µL XTT labeling mixture (Roche) per well for 4 h at 37 °C and 5% CO_2_. Absorbance of the formazan products was measured at 450 nm using Tecan’s Spark Microplate reader, while the reference wavelength was read at 650 nm.

### Colony formation assays

The ability of RNASET2 to regulate colony growth was assessed by in vitro colony formation assays. A total of 3 × 10^4^ RNASET2-overexpressing or control PC-3 or 22Rv1 cells were seeded into a six-well plate and cultured for 10 days. Colony formation was detected following fixing with 4% paraformaldehyde (PFA) and staining with 500 µL of crystal violet solution for 20 min at room temperature with gentle shaking. After three washes in distilled water, plates were allowed to dry for 2 h at room temperature. Finally, cell-retained crystal violet was dissolved in 500 µL of crystal violet elution buffer (50% ethanol and 0.1% acetic acid) and 100 µL of the solution was transferred to a six-well plate for final measurement. Colony formation efficiency was assessed by measuring absorbance at 595 nm using a SpectraMax M2 a microplate reader (Molecular Devices, Sunnyvale CA).

### Cell adhesion assays

RNASET2-overexpressing or control PC-3 or 22Rv1 cells (10^5^) were seeded into 24-well plates pre-coated with 2 ug/mL of Fibronectin (Sigma Aldrich) and cultured for 90 min at 37 °C in complete or serum-free RMPI 1640 (Euroclone). Following 90 min of incubation, cells were washed with PBS, fixed with 4% paraformaldehyde (PFA), and stained with 1 µg/mL DAPI (Sigma Aldrich). Cell adhesion was determined by counting cells in three random fields per well for each experimental condition, using a Zeiss Microscope associated with a Nikon camera.

### Immunofluorescence

To determine the role of RNASET2 in cytoskeletal organization in our experimental models, RNASET2-overexpressing cells and their RNASET2-null control counterparts from both 22Rv1 and PC-3 prostate cancer cell lines were examined in vitro by co-immunofluorescence assays using a FITC-labelled anti-RNASET2 antibody coupled to TRITC-phalloidin staining. Briefly, cells were grown on coverslips for 48 h, fixed with 4% paraformaldehyde and permeabilized with 0.1% Tween-20. After blocking with 2% Bovine Serum Albumin (BSA), cells were incubated with fluorescent ligands in a solution of 2% BSA and 0.1% Tween-20 in PBS. Coverslips were mounted onto microscope slides. Images were captured using a Leica TCS SP5 X confocal laser scanning microscope at a resolution of 1024 × 1024 pixels for 22RV1 and 913 × 913 pixels for PC-3 cells. The data were analyzed using ImageJ software (Rasband, W.S., U.S. National Institutes of Health, Bethesda, Maryland, USA).

### Cell migration assays

The ability of RNASET2 to modulate migration properties of PCa cell lines was investigated using a modified Boyden Chamber system. A total of 25 × 10^3^ RNASET2- overexpressing or empty vector-transfected PC-3 or 22Rv1 cells were added to the upper compartment of the Boyden chamber. An 8 μm pore-size polycarbonate filter coated with fibronectin (2 µg/mL, Sigma Aldrich) was used as an interface between the upper and the lower chambers for the migration assay. Complete RMPI 1640 medium was placed in the lower chambers, to induce FBS-directed migration, while serum-free complete RMPI 1640 medium was used as basal control. Following 6 h of incubation at 37 °C in 5% CO_2_, the Boyden chamber was disassembled, filters were recovered, washed in PBS, fixed in 4% PFA, and stained with DAPI (10 µg/ml; Sigma Aldrich), for 5 min at room temperature protected from light. Migrated cells were counted in a double-blind manner in 5 consecutive fields per filter, using a Zeiss Microscope associated with a Nikon camera.

### Real-time PCR

Total RNA was extracted from RNASET2-expressing and negative control (empty vector) PC-3 and 22Rv1 PCa cells using Trifast RNA extraction (Euroclone). RNA samples were treated with DNase (DNA-free Kit DNAse Treatment - Ambion) and reverse-transcribed into complementary DNA (cDNA) with random primers according to the instructions of the High-Capacity cDNA Reverse Transcription Kit (Applied Bioystems). Quantitative PCR was performed using gene-specific primers (Supplementary Table 1) on CFX Connect instrument (Biorad) and the Power SYBR-green PCR Master Mix (Applied Biosystems), following the manufacturer’s instructions. Each amplification reaction was performed in duplicate, and results were normalized using the housekeeping gene 18 S and showed as fold change over the average values of Empty PCa cells.

### Generation of conditioned media

Conditioned media (CM) was collected from RNASET2-expressing PC-3 and 22Rv1 PCa cells and their empty transfected control cells, following 72 h of incubation in RPMI 1640 medium, supplemented with 10% fetal bovine serum (FBS), 2 mM L-glutamine, 100 U/ml penicillin and 100 µg/ml streptomycin, at 37 °C, 5% CO_2_.

### Secretome analysis on antibody-membrane array

Conditioned media from RNASET2-expressing PC-3 and 22Rv1 PCa cells, as well as from their empty vector-transfected control cells lines were analyzed for soluble factor content using the C-series Human Cytokine Antibody Array C100, specifically the Human Cytokine Array C6 (RayBiotech, Inc., Norcross, GA, USA), as in [35]. After incubation, chemiluminescent signals were generated by HRP and detected with the Alliance Q9 instrument (Uvitec). Signal quantification was performed using ImageJ software and “Dot blot analyzer” plugin, version 1.53q, from the National Institutes of Health.

### Transmission electron microscopy

For ultrastructural analysis, pellets of PCa cells, following a wash in PBS, was fixed for 2 h at 4 °C in a mixture of 2.5% paraformaldehyde (PFA) and 2% glutaraldehyde in 0.05 mol/L, pH 7.3, cacodylate buffer, postfixed in 1% osmium tetroxide and embedded in epon-nma (EMBed-812 EMS). Thin sections were counterstained with uranyl acetate and lead citrate and examined with a JEOL1400Plus transmission electron microscope (CRIETT Center of University of Insubria). Three pellets of 22Rv1 empty, of 22Rv1 RNASET2, of PC-3 empty and of PC-3 RNASET2 cells, from independent experiments, were analyzed. For each pellet analyzed, 100 cells were considered and used to inspect the state of the Golgi apparatus and RE, as readout of hypertrophy (showed as % of cells), associated with increased secretory activities.

### Western blotting

For RNASET2 level evaluation, RNASET2 overexpressing or empty PC-3 or 22Rv1, parental THP-1 and RNASET-silenced THP-1 cells were lysed in RIPA buffer supplemented with protease and phosphatase inhibitor cocktails (Roche Diagnostics GmbH). Total protein quantification was performed with Bradford reagent (BIO-RAD), using bovine serum albumin as standard. Conditioned media were collected for all cell lines used, and used to detect secreted RNASET2 levels, as soluble factor.

For the SDS-PAGE analysis, 60 µg of intracellular lysate were loaded for each lane. Alternatively, 50 µl of cellular conditioned media collected at 90–100% confluence was loaded for each lane. Proteins from cell lysates or conditioned media were separated on 13% Bis-Tris Gel composed as follow: Stacking Gel (H_2_O, TrisHCl 1,5 M pH 6,8, Acrylamide solution 30% (PanReac AppliChem), glycerol 50%, SDS 20%, APS 10%, TEMED), Resolving Gel (H_2_O, TrisHCl 1,5 M pH 6,8, Acrylamide solution 30% (PanReac AppliChem), glycerol 50%, SDS 20%, APS 10%, TEMED) and transferred to a PVDF membrane Amersham Hybond (GE Healthcare Biosciences).

Membranes were incubated overnight at 4 °C with a blocking solution (5% powder milk resuspended in PBS-T). Membranes were subsequently incubated with the anti-human RNASET2 polyclonal antibody or anti-human Tubulin (T9026) and with peroxidase-linked anti-rabbit IgG secondary antibody (GE Healthcare Life science) or peroxidase-linked anti-mouse IgG secondary antibody (PAB10746, Abnova) for 1 h at room temperature. Specific band intensity was detected by Odissey FC (LI-COR) with a chemiluminescent substrate, WESTAR ETA C ULTRA 2.0, Cyanagen (Sigma Aldrich, St. Louis, MO, USA).

For cyclin D1 level evaluation, RNASET2 overexpressing or empty PC-3 or 22Rv1 cells were lysed in in RIPA buffer, supplemented with protease and phosphatase inhibitor cocktails (Roche Diagnostics GmbH). Proteins from cell lysates or conditioned media (30 µg of total protein) were separated on the NupageNovex on 4–12% Bis-Tris Gel (Life Technologies) and transferred to a PVDF membrane Amersham Hybond (GE Healthcare Biosciences). Membranes were incubated overnight at 4 °C with the anti-human β-Actin polyclonal antibody (1:1000) or anti-human cyclinD1 (E3P5S, 1:1000) and with peroxidase-linked anti-rabbit IgG secondary antibodies (GE Healthcare Life science 1:1000) for 1 h at room temperature. Specific band intensity was detected by the automated Alliance Q9 Advanced instruments (UVITEC).

### In vivo studies

We investigated the effects of RNASET2 overexpression in vivo using Nude Mice-Nu/Nu male mice (Charles River) xenografted with RNASET2 overexpressing PCa cell lines. RNASET2 overexpressing PC-3 and 22Rv1, as well as their corresponding empty vector controls (1 × 10^6^), were subcutaneously injected, with 100 µL of 10 mg/mL Matrigel (final volume of injection 300 µL) into the right flank of 5–6 weeks male nude mice. Five animals per group were used. Tumor cell growth was monitored twice a week by measuring tumors size with a digital caliper. Following 28 days post-injection, tumors were excised, imaged, and weighted. Parts of the excised tumors were mechanically processed to obtain a single-cell suspension for FACS analysis to assess macrophage polarization. Animal handling complied with the Italian and European Community guidelines (D.L. 2711/92 N°116; 86/609EEC Directive), the 3 Rs principles, and received approval from the institutional ethics committee. All the procedures applied were approved by the local animal experimentation ethic committee (ID# #06_16) of the University of Insubria and by the Health Ministry (ID#225/2017-PR).

### Clinical samples

Peripheral blood (PB) samples (12 ml of whole blood collected in EDTA) were obtained from individuals diagnosed with prostate adenocarcinoma. The control group (HC) consisted of peripheral blood from healthy, tumor-free male subjects. Exclusion criteria for the study included patients with diabetes, human immunodeficiency virus (HIV), hepatitis C virus (HCV), hepatitis B virus (HBV) infections, chronic inflammatory conditions, those undergoing chemotherapy or radiotherapy, those who were iatrogenically immunosuppressed, or those subjected to myeloablative therapies. The study protocol (No. 463.2021) was approved by the institutional review board ethics committees and adhered to the principles outlined in the Helsinki Declaration of 1975, revised in 2013. Informed consent was obtained from all participants, in compliance with the Helsinki Declaration of 1975, revised in 2013. Demographic and clinical features of the enrolled subjects are illustrated in Table 1 and 2.

**Table 1:** Patient’s clinical features

ID	Gender	AGE	Hystology	TNM	Prognostic grade	Gleason
PZ1	M	59	acinar adenocarcinoma	T2 N0	2	3 + 3
PZ2	M	71	acinar adenocarcinoma	pT3aN0R1	4	4 + 4
PZ3	M	65	acinar adenocarcinoma	pT2 N0	2	3 + 4
PZ4	M	74	acinar adenocarcinoma	pT3 N0	2	3 + 4
PZ5	M	68	acinar adenocarcinoma	pT2 N0	3	4 + 3
**Age**: 67,4 ± 5,77 (Mean ± SD)						

**Table 2:** Healthy controls’ clinical features

ID	GENDER	AGE
HC1	M	55
HC2	M	53
HC3	M	57
HC4	M	56
**Age**: 55,25 ± 1,7 (Mean ± SD)		

### Flow cytometry

A total of 5 × 10^5^ of total PBMCs per FACS tube were stained for 30 min at 4 °C with APC-conjugated anti -human CD14 (clone TUK4, Miltenyi Biotech). Following PBS wash, cells were stained with anti-human RNAET2 for 30 min at 4 °C. After a second wash, cells were stained with PE-conjugated anti-IgG secondary antibody (R&D System) for 30 min at 4 °C. Cells stained with PE-conjugated anti IgG secondary antibody alone were used as controls. Cells were analyzed using a BD FACS Fortessa x20 flow cytometer equipped with 5 lasers. Following Forward/Side Scatter setting, monocytes were identified by their morphological features and as CD14^+^ cells. RNASET2 expression was detected on CD14^+^ gated monocytes.

### In silico analysis and data set interrogation

Survival analyses were performed using the cSurvival online data repository (https://tau.cmmt.ubc.ca/cSurvival/). Tumor Immune Single-cell Hub (TISCH, http://tisch.comp-genomics.org/), was used to evaluate the expression of RNASET2 gene in two different datasets of prostate cancer (GSE141445 and GSE143791).

For in-silico analysis of monocytes and macrophages, single cell RNA-seq public gene expression data were obtained from the Gene Expression Omnibus (GEO), repository (https://www.ncbi.nlm.nih.gov/gds): GSE153892 [36] and re-analyzed as detailed below.

Samples from the GSE153892 dataset were analyzed following Seurat differential expression workflow [37]. Briefly, after loading samples into a Seurat object, the following filtering conditions were applied: number of genes detected per cell between 300 and 3,000; total number of molecules detected per cell < 15,000; percent of mitochondrial gene counts below 10%.

Raw counts were normalized through SCT transformation [37, 38]After dimensionality reduction, cell clusters were defined using the Louvain algorithm and a resolution = 0.6. Differentially expressed genes in relevant clusters were detected by the MAST algorithm implemented in Seurat.

TIMER2.0 (http://timer.cistrome.org/) online server was used to evaluate macrophage infiltration levels according to RNASET2 expression.

String analysis for the biological connections among the most upregulate soluble factors, in RNASET2-expressing PCa cells, as detected by secretome analysis of their related conditioned media.

### Direct co-cultures of THP-1 derived macrophages with PCa cells

THP-1-derived monocytes [control, a *RNASET2*-silenced pool and a RNASET2-silenced single clone (#50] [14] were plated on glass coverslips in 24-well plates at 0.7 × 10^6^ cells/ml in RPMI-1640 medium supplemented with 10% heat-inactivated FBS, 1% glutamine and 5 ng/ml Phorbol 12-myristate 13-acetate (PMA) for 48 h to induce differentiation into M0 macrophages. Thereafter, 10^6^ pcDNA-transfected, GFP^+^ 22Rv1 or PC-3 cells were added to each well to establish direct cell-to-cell contact in co-culture. Two independent GFP + clones were used for both PCa cell line: clones #2 and #3 for 22Rv1 cells and clones #10 and #12 for PC-3 cells. The amount of viable cancer cells was assessed following 24 h co-culture by counting the GFP^+^ fluorescent area in each well. Briefly, cells were fixed in 4% paraformaldehyde and permeabilized with 0.1% Tween-20. Coverslips were then mounted on microscope slides and several images were captured from each well using an Olympus IX51 fluorescence microscope with a scan format 2736 × 1824 pixels. Data were analyzed using ImageJ software.

### FITC-dextran endocytosis assay

THP-1 cells were differentiated in M0 macrophages using PMA for 48 h as described above. The day after, THP-1 macrophages were exposed to FITC-dextran particles (0.5 mg/ml) for 90 min at both 4° C (negative control) and 37° C. Cells were then briefly washed with warm PBS, detached, and transferred to FACS tubes. Following staining with a fixable viability dye (7-aminoactinomycin D) for 10 min at 4° C, cells were washed and fixed with 4% paraformaldehyde. Analysis was performed using ImageJ software with “Analysed Particles” plugin, following the conversion of the images to 8-bit greyscale.

### Statistical analysis

Data are expressed as means ± SEM. The statistical significance between two data sets was determined by the student t-test. Results from tumor growth curves were determined by two-way ANOVA. One-way ANOVA followed by Dunnett’s multiple comparison test was used for Secretome analysis. *P* values (p) ≤ 0.05 were considered statistically significant. Data were analyzed using the GraphPad Prism9 (San Diego, CA). Flow cytometry data were analyzed using the BD FACS-Diva and the FlowJo-v10 software. For survival analysis, Chi-squared statistic was used to assess the differences in PFI between RNASET2^high^ vs. RNASET2^low^ patients.

### Image quantification tools

EBImage was used to obtain masks of the GFP fluorescence emitted by cells [39]. Since no additional measurements were available at the time, to aid the cell identification process (e.g., co-staining of nuclei), no segmentation of individual cells was attempted. Instead, the entire fluorescent area was captured by means of adaptive thresholding, using a 100 × 100-pixel window and an offset value of 0.1 (function *thresh(image*,* width*,* height*,* offset)* from EBImage). The entire fluorescent area was calculated per image (n_images_=240), and Mann Whitney U/Kruskall Wallis test was employed for contrasts between two or more groups respectively. To ensure that cell autofluorescence was not introducing bias to our measurements, the procedure was also repeated with a range of offset values (i.e., 0.005, 0.01, 0.05, 0.2, 0.3, 0.5, 0.7, 0.9), resulting in differing stringency in area capture. Contrasts achieving significance with *p* < 0.05 were considered significant.

## Results

### RNASET2 expression in patients with prostate cancer is associated with an improved progression-free interval

**Fig. 1:**
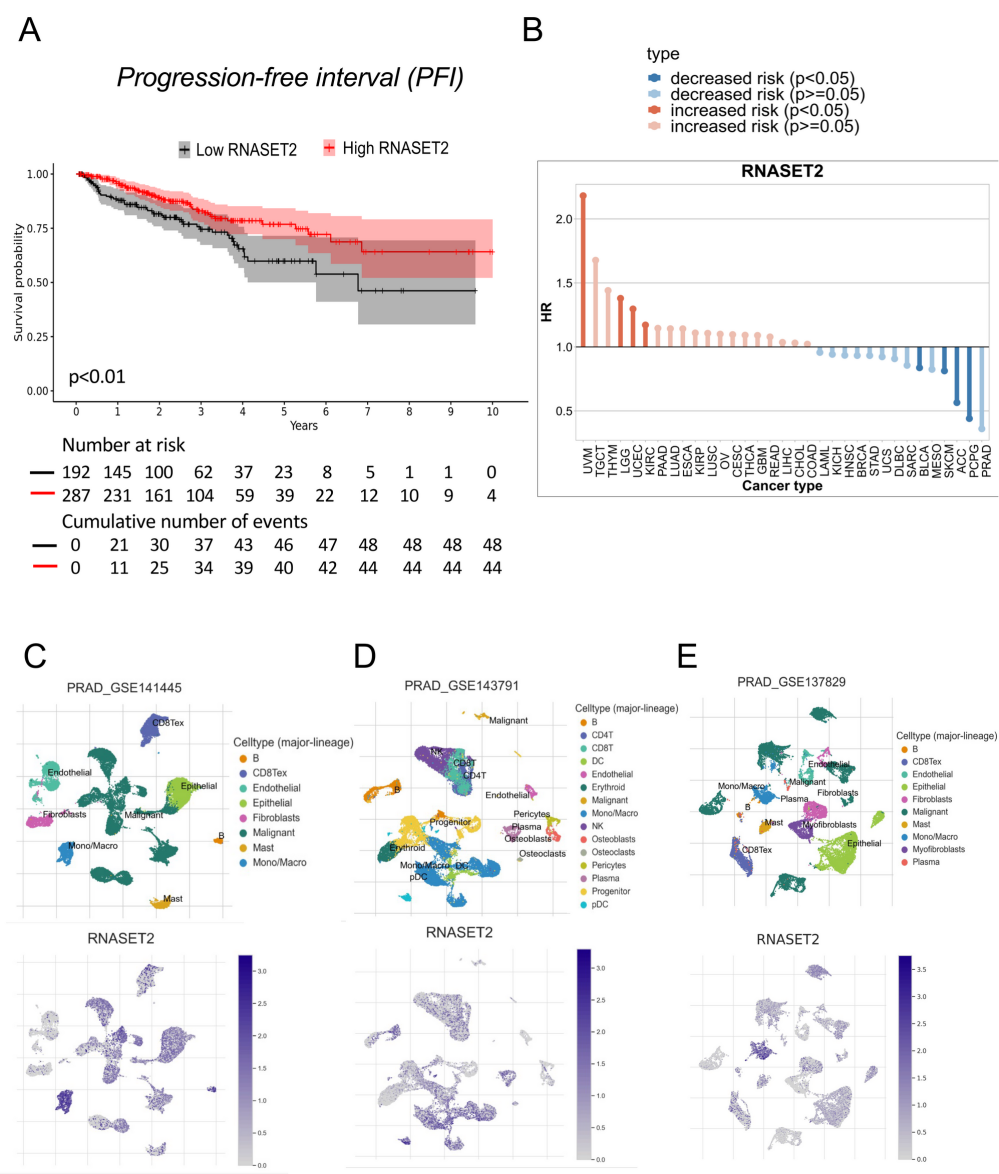
Clinical impact of *RNASET2* expression in patients with prostate cancer. Clinical impact of RNASET2 expression in patients with prostate cancer. Kaplan–Meier curves showing the progression-free survival of patients with prostate cancer dichotomized according to RNASET2 expression (cutoff 40%). Survival analysis was performed using cSurvivalv1.0.1 online bioinformatic tool based on TCGA project **(A).** Survival analysis performed using TISCH2 (Tumor Immune Single-cell Hub 2) online portal showed that high expression of RNASET2 correlates with a decreased risk in patients with prostate cancer (**B**). Comparative evaluation of three distinct single-cell RNA-seq datasets GSE141445 (**C**), GSE143791 (**D**), GSE137829 (**E**), performed using TISH2 portal, showing the expression of RNASET2 in patients with prostate cancer

To evaluate the clinical impact of *RNASET2* expression levels on the survival of prostate cancer (PCa) patients, we performed an in-silico analysis using the cSurvival online tool, using available datasets. Patients with high expression of RNASET2 levels showed a significant improvement in progression-free interval (PFI), compared to those with lower RNASET2 expression levels (Fig. 1A). To further corroborate the potential protective role of RNASET2 in PCa, we used the TISCH2 software to analyze additional datasets. Our analysis indicated that, across various tumor types, RNASET2 expression was associated with a trend for a reduced risk in PCa, even if this association did not reach statistical significance (*p* ≥ 0.05) (Fig. 1B).

Furthermore, a comparative analysis of three distinct single-cell RNA-seq data sets revealed that RNASET2 is predominantly expressed by tumor microenvironment cells, particularly immune cells, fibroblast, and epithelial cells. Conversely, a lower expression in malignant cells was detected. This finding aligns with RNASET2’s proposed to function as a tumor suppressor gene (TSG) and its cell-autonomous oncosuppressive role in the TME (Fig. 1C-E). These observations were further supported by data from the Human Protein Atlas (https://www.proteinatlas.org/), prompting us to test the effects of RNASET2 overexpression in PCa cell lines.

### RNASET2 impairs cell proliferation and colony formation in 22Rv1 cells

We next investigated the effects of RNASET2 overexpression on cell proliferation in two distinct PCa cell lines, namely PC-3 and 22Rv1. These cell lines were selected following a comprehensive evaluation of RNASET2 expression levels across commonly used PCa in vitro models, using the Protein Atlas Database as a reference (Supplementary Fig. 1). This analysis revealed that both 22Rv1 and PC-3 cell lines display low endogenous levels of RNASET2 expression. Additionally, these cell models reflect distinct characteristics of human PCa tumors: 22Rv1 cells represent prostate adenocarcinomas, while PC-3 cells resemble small-cell neuroendocrine carcinomas, an aggressive PCa variant. We first confirmed the null RNASET2 expression in both cell lines, by western blot analysis, in cell extracts and supernatants (Supplementary Fig. 1B-C), before proceeding with RNASET2 overexpression in these cells, which was verified and confirmed in both cell extracts and supernatants (Supplementary Fig. 2A-C).

**Fig. 2:**
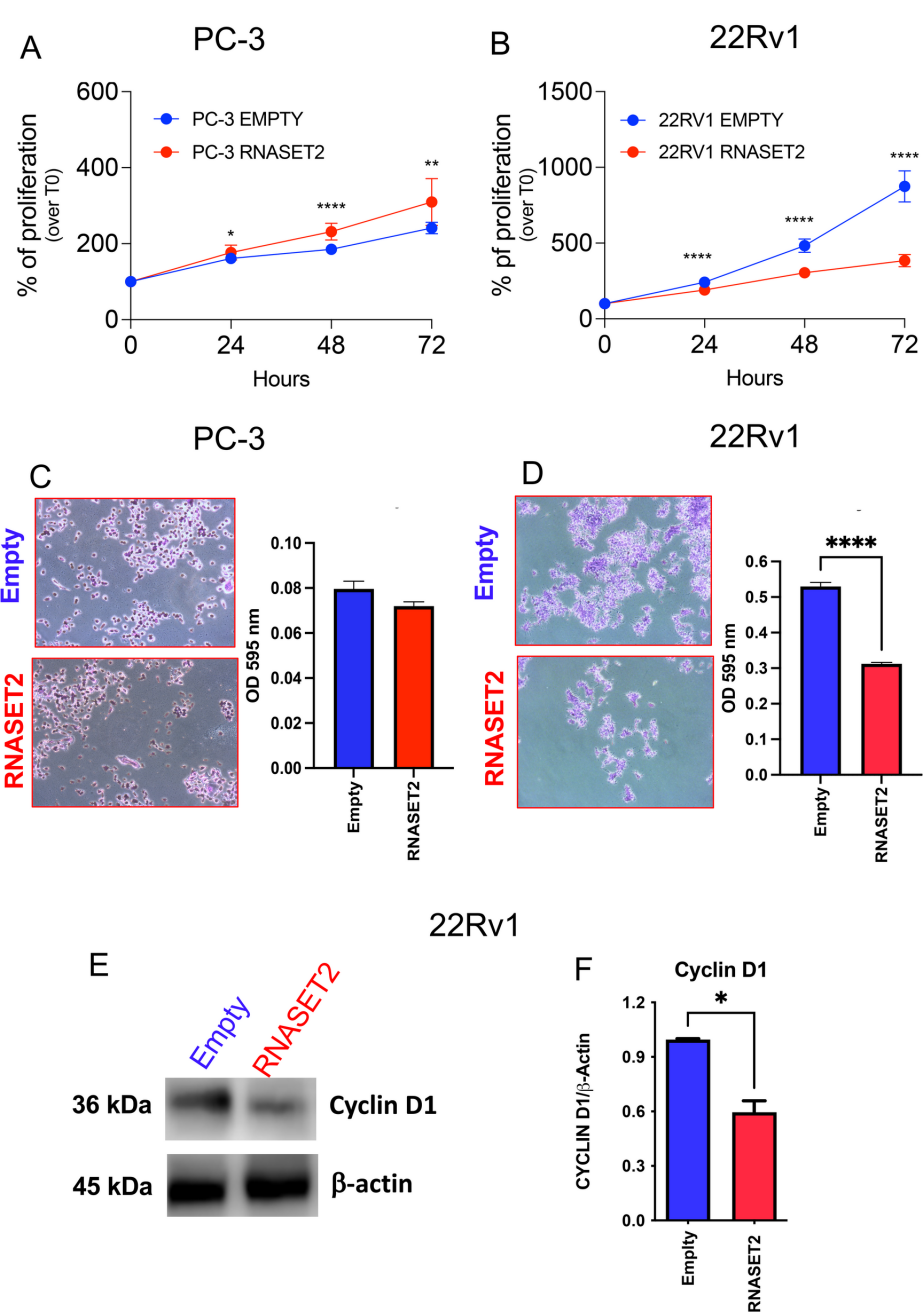
Effects of RNASET2 on PCa cell proliferation. The effect of RNASET2 overexpression of cell proliferation was assessed by crystal violet assay (*N* = 2 in dodecuplicate) and colony formation assay (*N* = 3 in triplicate) on PC-3 and 22Rv1 prostate cancer (PCa) cell lines. The proliferation rate was monitored for 24-48-72 h in RNASET2 overexpressing PC-3 (**A**) and 22Rv1 (**B**) cells, as compared to empty control. Graphs and representative images showing the effect of RNASET2 overexpression on PC-3 and 22Rv1 on their ability to generate colonies (**C-D**, magnification 10x), following 10 days of cell culture. Western blot was used to determine the modulation of the CyclinD1 molecule (**E**), in RNASET2-overespressing 22Rv1 cells and compared to empty controls. Data are showed as mean ± SEM, Two-way ANOVA (for growth curves), t-student test (for colony-formation assay and western blot), *p < 0.05, ***p < 0.001, ****p < 0.0001

We then evaluated the effect of RNASET2 expression in the two PCa cell lines and observed that RNASET2-overexpressing PC-3 cells exhibit a weak but statistically significant increase in proliferation rate, compared to control PC-3 cells transfected with the empty vector (Fig. 2A). By contrast RNASET2-overexpressing 22Rv1 cells showed a marked and statistically significant reduction of cell proliferation, compared to their empty-vector counterpart (Fig. 2B). Accordingly, RNASET2-expressing 22Rv1 cells showed a decreased capability to generate colonies, compared to empty vector-transfected 22Rv1 cells; this effect was not observed in RNASET2-overexpressing PC-3 or empty vector-transfected PC-3 cells (Fig. 2C-D). Consistently, western blot analysis revealed that the significantly reduced proliferation rate of RNASET2-expressing 22Rv1 was associated with a statistically significant decreased levels of the cell cycle regulator Cyclin D1, compared to empty 22Rv1 cells (Fig. 2E).

### Cell adhesion and migration patterns are differentially affected by RNASET2 overexpression in PC3 and 22Rv1 cells

**Fig. 3:**
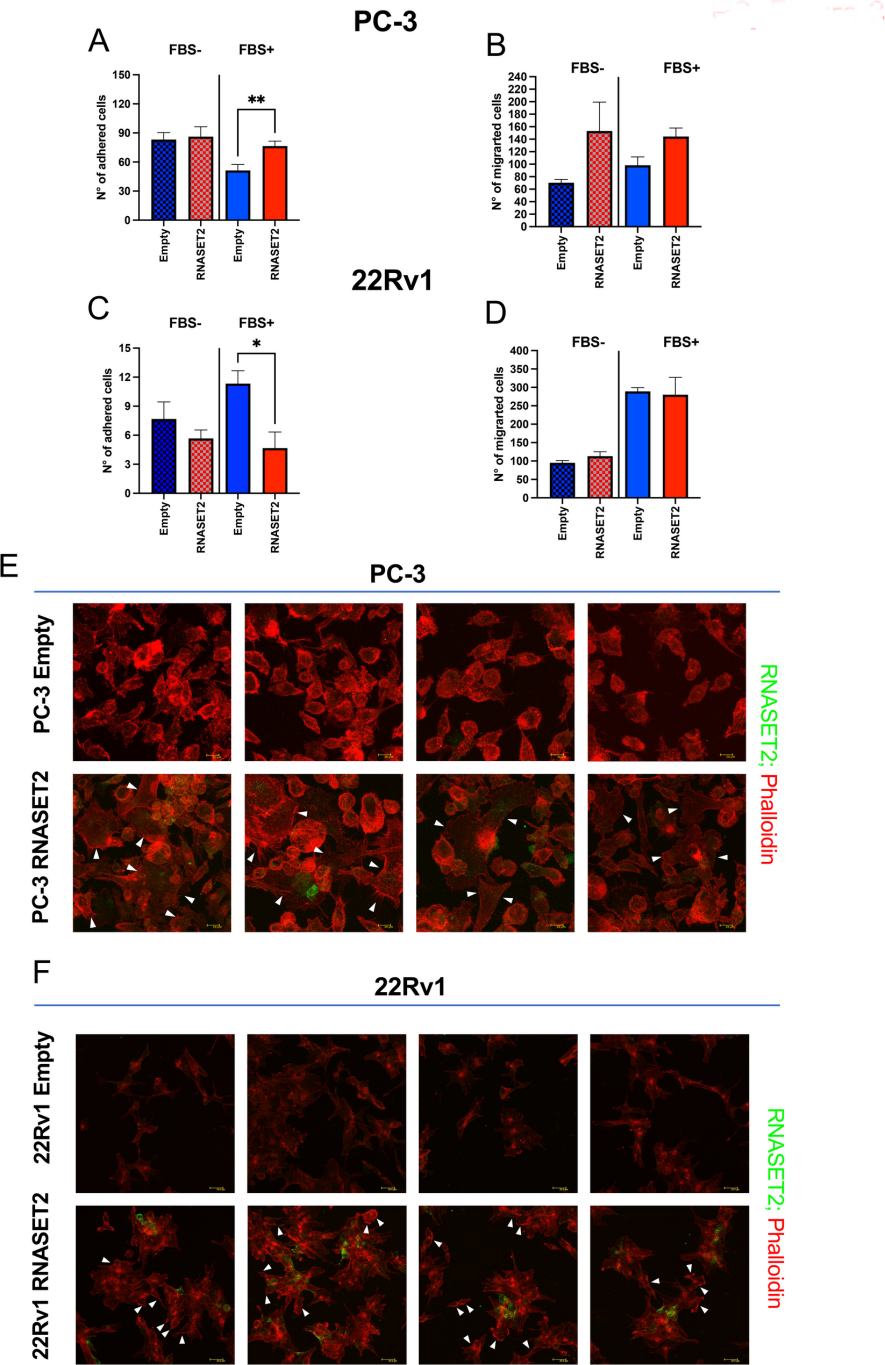
Effects of RNASET2 on cell adhesion migration and cytoskeleton modification in PCa cell lines. The effect or RNASET2 expression on key metastatic features of PCa cells was investigated: adhesion assay **(A**,** B**) (*n* = 3, in triplicate), migration assay (*n* = 2, in triplicate), in presence or absence of FBS as driving stimulus **(C**,** D**) and cytoskeleton morphological analysis (*n* = 4), with white arrowheads pointing at cell spreading processes observed in PC-3 cells and actin clustering at the cell periphery in 22Rv1 cells, respectively **(E**,** F).** RNASET2 (green) and Phalloidin (red) staining are shown. Data are showed as mean ± SEM, t-student test, *p < 0.05; **p < 0.01. E: empty control cells; T2: RNASET2-overexpressing cells

We next investigated the contribution of RNASET2 expression on the modulation of key features of the metastatic behavior in PCa tumor cells, specifically cell adhesion and migration. We found that RNASET2-expressing PC-3 cells acquire increased capabilities to adhere to a fibronectin layer, in a statistically significant manner, in presence of FBS (Fig. 3A), as an inducer of cell adhesion. By contrast, RNASET2-expressing 22Rv1 cells exhibited a reduced ability to adhere to fibronectin, both in presence and absence of FBS (Fig. 3C). Additionally, while RNASET2-overexpressing PC-3 cells showed a trend towards increased migration, compared to PC-3 empty-vector transfected cells, regardless of FBS presence (Fig. 3B), RNASET2-overexpressing and empty-vector transfected 22Rv1 cells displayed similar migration activity (Fig. 3D).

### RNASET2 impacts on cytoskeletal organization in PCa cell lines

To evaluate whether the above-mentioned RNASET2-mediated changes in cell behavior might be partially associated with RNASET2’s established role in cytoskeletal reorganization, RNASET2-expressing 22Rv1 and PC-3 cells, along with their respective empty-vector controls, were stained with phalloidin-TRITC, a mycotoxin used to visualize actin filaments in cell cultures, and a FITC-labeled anti-RNASET2 antibody (Fig. 3E-F). In PC-3 cells, RNASET2 overexpression triggered a marked change in cell morphology, characterized by extensive spreading of several cells on the culture vessel surface, consistent with increased cell adhesion and potentially the formation of migratory lamellipodia or filopodia (Fig. 3E). In contrast, RNASET2 expression in 22Rv1 were associated with a different pattern, characterized by cell aggregation in clusters, coupled to the appearance of actin bundles at the cell periphery without apparent changes in cell size (Fig. 3F). These contrasting patterns likely correspond to the previously described differential adhesion or migration rates.

### RNASET2 decreases the expression of key genes involved in epithelial-to-mesenchimal transition, metastasis and immunesupression

**Fig. 4:**
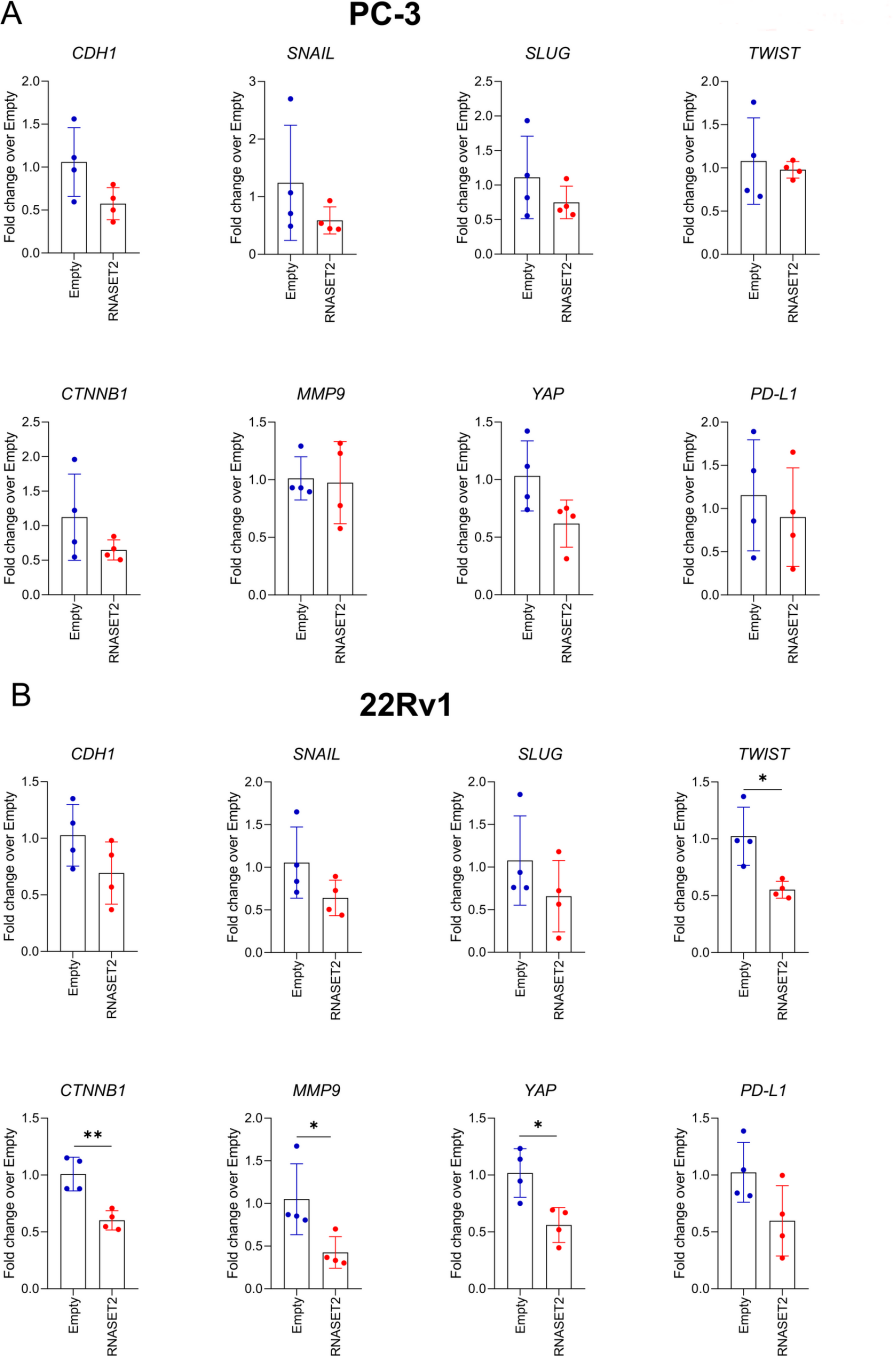
Molecular analysis on pro-tumor factors expression in RNASET2-overexpressing PCa cell lines. Graphs show the effects of RNASET-overexpressing and empty control PC-3 (**A**) and 22Rv1 (**B**) PCa cell lines, as detected by real-time PCR on *CDH1*,* SNAIL*,* SLUG*,* TWIST*,* CTNNB1*, (markers of EMT), *MMP9*,* YAP* (markers of metastasis) and *PD-L1* (marker of immunosuppression expression. Data are shown as mean ± SEM, Student T-test, *p < 0.05; **p < 0.01

We also tested the effects of RNASET2 overexpression on key genes involved in epithelial-to-mesenchimal transition (EMT), metastasis and immunesuppression. Once again, we observed different effects when comparing PC-3 and 22Rv1 cells (Fig. 4A-B). In RNASET2-overexpressing 22Rv1 cells, we found decreased expression, even though not statistically significant, of *CDH1*,* SNAIL*,* SLUG*,* TWIST*,* CTNNB1*, (markers of EMT), *MMP9*,* YAP* (markers of metastasis) and *PD-L1* (a marker of immunesuppression) compared to empty-vector 22Rv1 cells (Fig. 4B). This effect was not not fully replicated in PC-3 cells, where only *CDH1*,* SLUG*,* CTNNB1*, and *YAP* showed decreased expression, though also not statistically significant, when comparing RNASET2-overexpressing PC-3 cells to empty vector PC-3 cells (Fig. 4A).

### Effects of RNASET2 on the modulation of soluble factor production by PCa cell lines

**Fig. 5:**
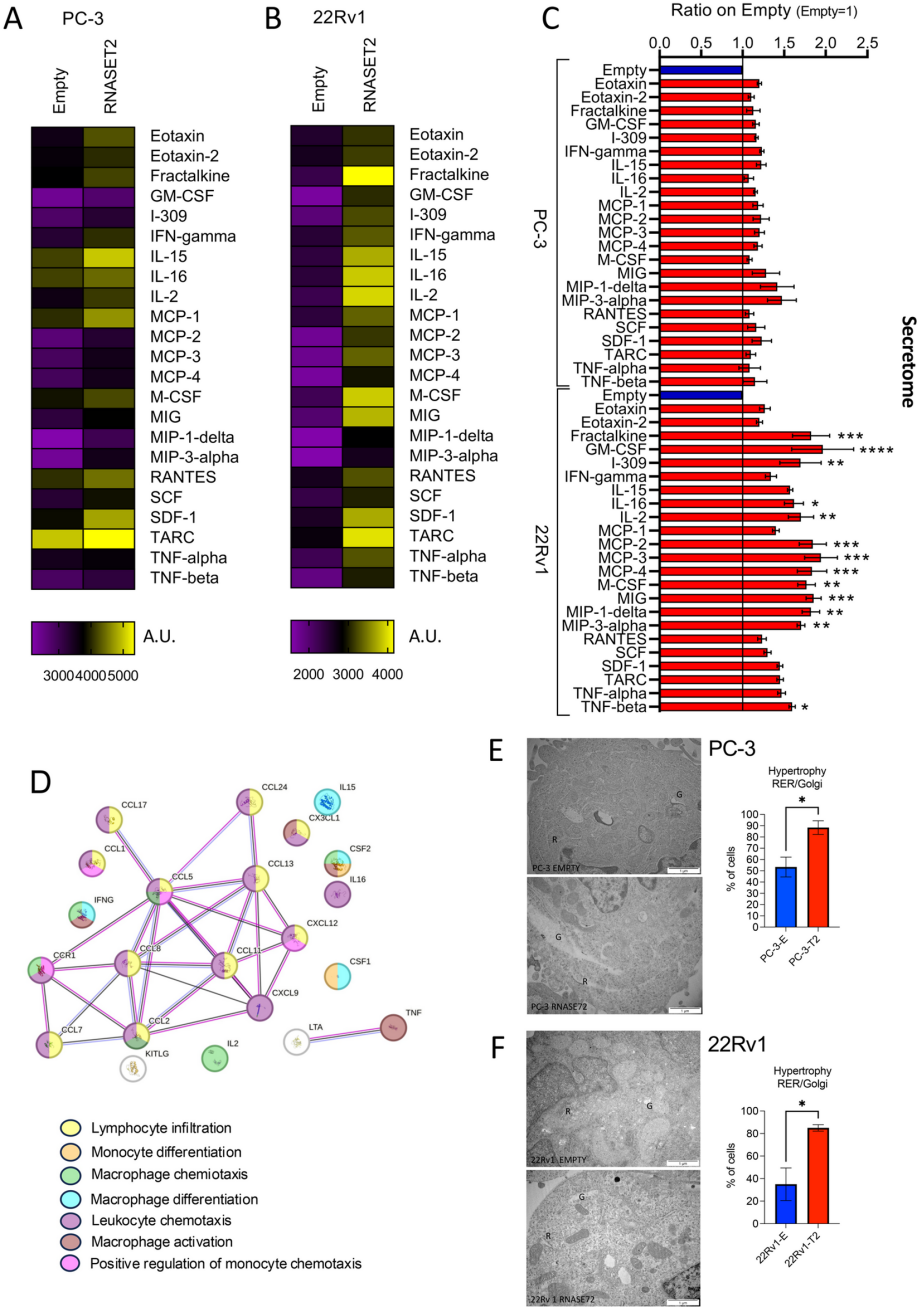
Secretome analysis of soluble factors released by RNASET2-empty and RNASET2-overexpressing PC-3 and 22Rv1 cells. Heatmap showing the modulation of the main immunomodulatory factors involved in monocyte/macrophage recruitment and polarization in the conditioned media from RNASET2-empty vector and RNASET2-overexpressing PC-3 cells (**A**) and RNASET2-empty vector and RNASET2-overexpressing 22Rv1 cells (**B**). Bar plot showing the modulation of the selected soluble factors by PC-3 and 22Rv1 cells overexpressing RNASET2 represented as fold change over the respective empty controls (**C**). String analysis for the biological connections among the most upregulate soluble factors, in RNASET2-expressing PCa cells, as detected by secretome analysis of their related conditioned media (**D**). Transmission electron microscopy (TEM) of PC-3 and 22Rv1 RNASET2 overexpressing and empty control PCa cells, and related graphs showing the quantification of Reticulum (R) and Golgi (G) hypertrophy. Data are shown as mean ± SEM, One-way ANOVA, *p < 0.05; **p < 0.01; ***p < 0.001, ****p < 0.0001, or mean ± SEM student t-test *p < 0.05

We then turned to evaluate the effects of RNASET2 modulation in regulating the release of soluble factors by 22Rv1 and PC-3 PCa cell lines (Supplementary Fig. 3). We observed that both RNASET2-overepressing PC-3 and 22Rv1 PCa cells are characterized by increased secretion of soluble factors involved in monocyte/macrophage recruitment and activation, compared to their empty-vector transfected control cells (Fig. 5A-D). Comparing the two cell lines, we observed that only RNASET2-overexpressing 22Rv1 showed a significantly increased release of soluble factors biologically involved in leucocyte infiltration/chemotaxis, including monocytes and macrophages, monocyte and macrophage activation and differentiation (Fig. 5A-C).These include MCP family member proteins, M-CSF, MIP-1delta, and TNF-beta, but also cytokines/chemokines, such as IL-2, IL-15, IL-16, fractalkine (CX3CL1), GM-CSF, MIG, IFNγ involving both innate (NK, DC) and adaptive (T) cells and their potential antitumor activities (Fig. 5B, C). By contrast, RNASET2-overepressing PC-3 cells displayed only a limited increase in these factors (Fig. 5B, C). This suggests that RNASET2-overepressing 22Rv1 cells might strongly shape a different macrophage-oriented and tumor suppressive TME, compared with RNASET2-overepressing PC-3 cells, in turn possibly impacting on prostate cancer cell growth in-vivo.

An electron transmission microscopy analysis showed that RNASET2-overexpressing PC-3 and 22Rv1 cells exhibited a morphological pattern of increased secretion, as detected by the increased hypertrophy of reticulum cisternae with a well-developed Golgi apparatus, as readout of augmented protein synthesis, compared to their respective empty control cells (Fig. 5E-F).

### RNASET2 affects cell growth and macrophage polarization of 22Rv1 cells in vivo

**Fig. 6:**
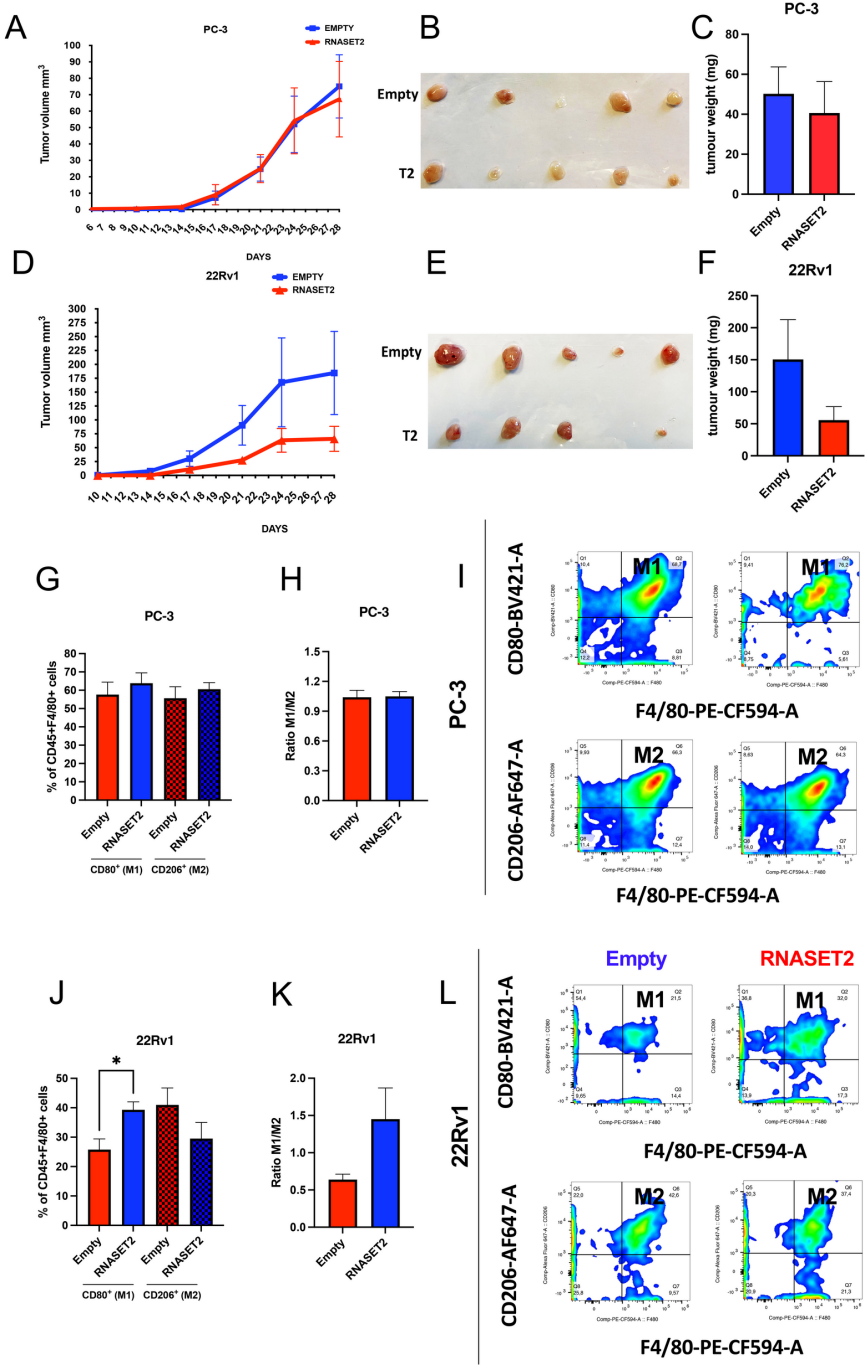
Effects of RNASET2 on tumor cell growth in vivo. The effects of RNASET2-overxpression in PCa cells were investigated on tumor cell growth in vivo. Nude Mice-Nu/Nu male mice (*n* = 5) were subcutaneously injected with RNASET2-overexpressing. Growth curve graphs **(A**,** D**), photographs of excised tumors **(B**,** E**), and tumor weight **(C**,** F**) of RNASET2-overexpressin PC-3 and 22Rv1 PCa tumor xenografts, and their related empty-vector transfected controls, are shown. FACS analysis (*n* = 3 animals/group) for tumor-infiltrating CD80^+^ M1 and CD206^+^ M2 macrophages **(G**,** J**), M1/M2 ratio (**H**, **K**), and representative dot plots **(I**,** L**) in excised RNASET2-overexpressing PC-3 and 22Rv1 PCa xenograft, and their related empty-vector transfected controls. Data are showed as mean ± SEM, Two-Way ANOVA (for growth curves) and One-Way ANOVA (for flow cytometry results)

We next moved to an in vivo model to confirm the results on the effects of RNASET2 overexpression previously observed in vitro in the same human PCa cell lines, focusing on macrophage recruitment and polarization. We found that both *RNASET2-*overexpressing and empty-vector control PC-3 cells exhibit similar tumor growth (Fig. 6A-B) and weight (Fig. 6C) in vivo. Conversely, we observed that *RNASET2*-overexpressing 22Rv1 cells generate smaller tumors in terms of both growth rate (Fig. 6D-E) and tumor weight (Fig. 6F), compared to the empty-control counterpart. These results are consistent with those previously observed in vitro (Fig. 2). Furthermore, while no effects on macrophage polarization were detected in *RNASET2*-overexpressing vs. empty vector control PC-3 tumor-bearing mice (Fig. 6G-I), tumor samples collected from *RNASET2*-overexpressing 22Rv1 cells showed a statistically significant increase in M1-like macrophage infiltration, coupled with a trend toward decreased M2-like macrophage infiltration, as revealed by flow cytometry both in terms of percentage of M1 and M2-like macrophages and M1/M2 ratio (Fig. 6J-L).

### In silico analysis shows that RNASET2 is down-regulated in PCa macrophages and monocytes

**Fig. 7:**
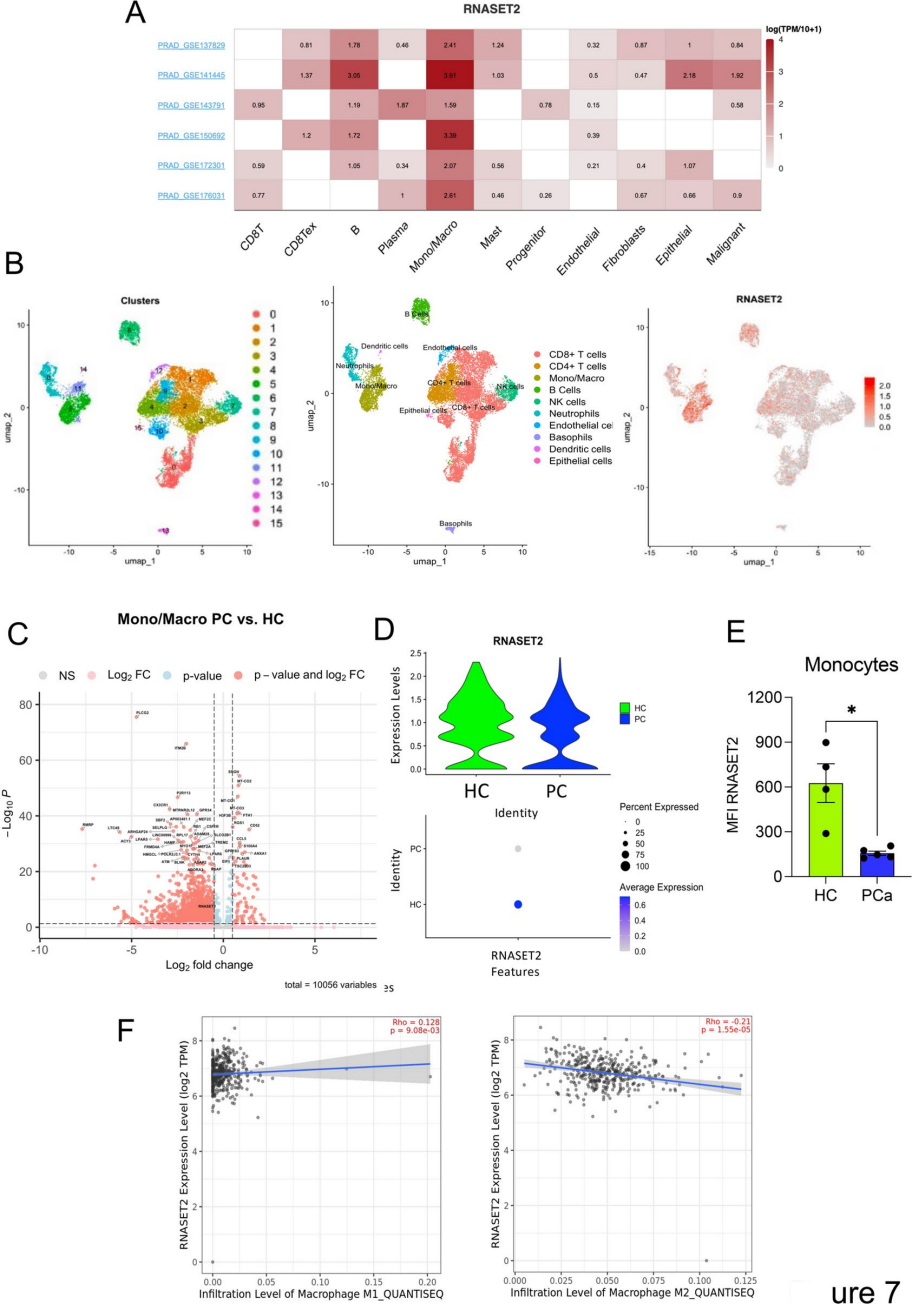
RNASET2 expression is down-regulated in monocytes and macrophages derived from prostate cancer samples compared with normal tissues. TISCH2 analysis of RNASET2 expression among the tumor microenvironment components of prostate cancer in 6 different datasets (GSE141445, GSE143791, GSE137829, GSE150692, GSE172301, and GSE176031) **(A).** GSE153892 [36] dataset showing CD45^+^ cells sorted from human prostatic tumoral (*n* = 3) and non-tumoral tissues (*n* = 3) was re-analyzed. UMAP-based 2D visualization describing cell clusters and distinct cell types represented by different color codes are shown together with overall RNASET2 expression (**B**). Volcano plot displays the results of differentially expression analysis and shows the up-regulated and down-regulated genes in prostate cancer patients’ tissues (PC) and non-tumoral tissues (HC) (**C**). To assess the different expression of RNASET2, samples were divided in non-tumoral tissues (HC) and prostate cancer tumoral tissues (PC) (**D**). Increased expression of RNASET2 in monocytes from healthy control subjects compared to monocytes from PCa patients, a detected in our small cohort of subjects, by flow cytometry (**E**). Correlation plot generated using the TIMER2 software to interrogate the relation between RNASET2 expression and M1 or M2 macrophage infiltration in PCa tumor tissues (**F**)

Based on our in vivo results, we used the same online tool above described to compare five different single cell-RNA-seq datasets of PCa, and we observed that among the TME components, monocytes and macrophages expressed higher levels of RNASET2, compared to other cells within the PCa microenvironment (Fig. 7A). Thus, we focused on these cells to re-analyze a publicly available dataset (GSE153892 [36]) of CD45^+^ cells sorted from human prostatic tumoral (PC; *n* = 3) and non-tumoral tissues (HC; *n* = 3). (Fig. 7B-D). Focusing on monocyte/macrophage cluster, higher levels of *ADORA3*,* CX3CR1*,* MEF2A*,* ATM*, and *PLCG2* were found in HC compared to PC (Fig. 7C, Supplementary Table 2). Conversely, *ANXA1*,* CCL5*,* CD52*, and *S100A4* were up-regulated in PC macrophages (Fig. 7C, Supplementary Table 2). Among others, *RNASET2* levels were found to be down-regulated in monocyte/macrophage cluster of PCa patients in comparison with non-tumoral tissues (adj_*p* value = 7,6E-10) (Fig. 7D, Supplementary Table 2). To further validate these in silico observations, we evaluated RNASET2 expression levels in circulating monocytes, in blood samples of a small cohort of PCa patients and healthy subjects, by flow cytometry (Fig. 7E). In keeping with the in silico-derived data, we observed reduced levels of RNASET2 in monocytes derived from PCa patients, compared to those from healthy subjects, supporting data from sc-RNA-seq.

To test the possible correlation between RNASET2 levels and macrophage polarization in human prostate cancer tissue samples, we interrogated the TCGA dataset, using the publicly available portal TIMER2.0; This analysis showed a statistically significant, though moderate, direct correlation between RNASET2 levels and M1 macrophage infiltration in PCa patients. Conversely, an inverse correlation was observed between RNASET2 and M2 infiltration (Fig. 7F).

### RNASET2-silenced macrophages are impaired in their antitumoral activity and phagocytic activities

**Fig. 8:**
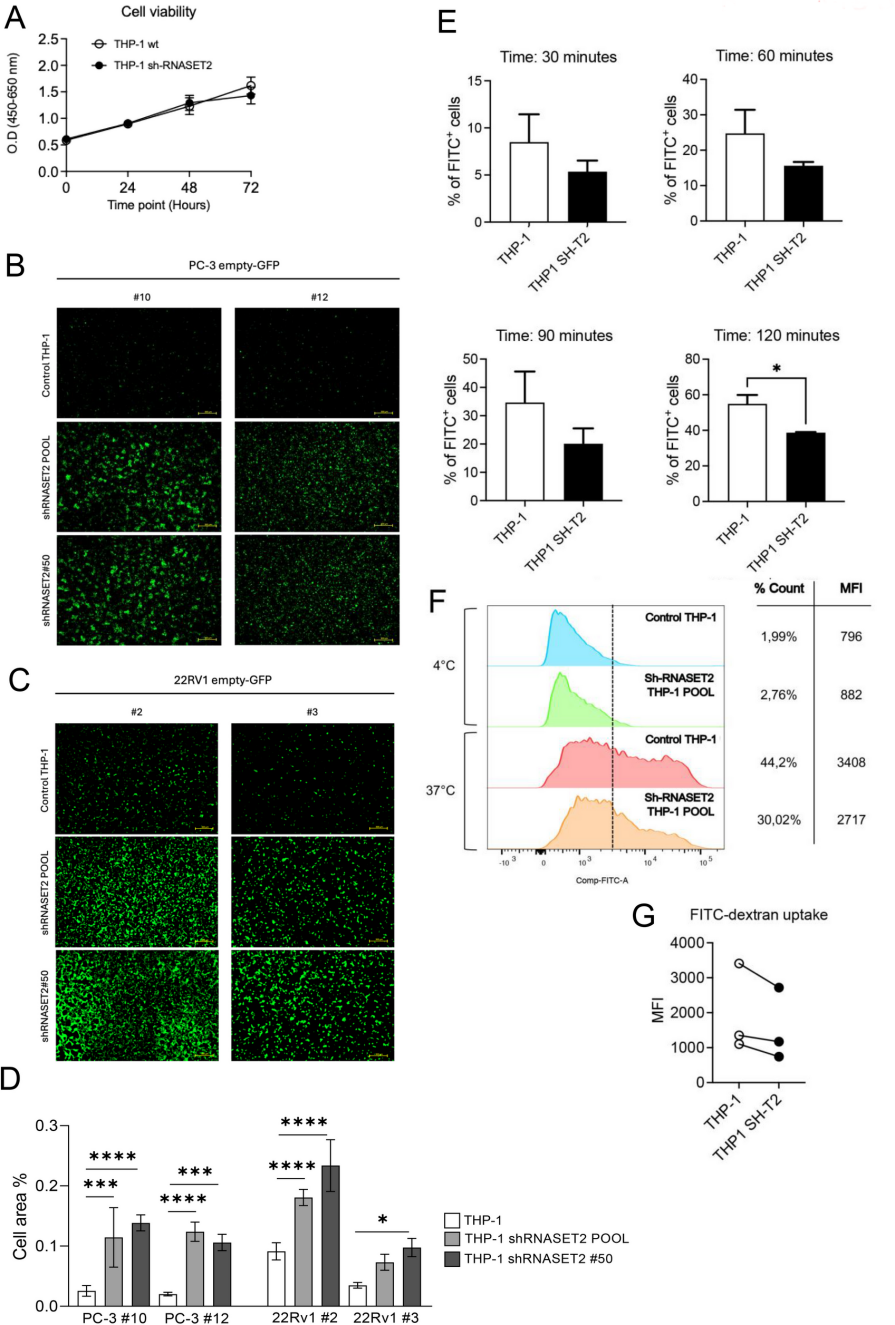
Effects of RNASET2 on anti-tumor activity and phagocytosis in THP-1 macrophages. Graphs illustrating the proliferation rate of wt of RNASET-2 silenced THP-1 cells (**A**). Representative images from immunofluorescence assay of PC-3 GFP^+^ clones #10 and #12 (**B**) and 22Rv1 GFP^+^ clones #2 and #3, (**C**) grown in co-culture with control, *RNASET2-*silenced pool and *RNASET2*-silenced (clone #50) THP-1 derived M0 macrophages (*N* = 3). Quantification of the % of green fluorescent area both for PCa clones was determined by XX **(D).** Data are shown as mean ± SEM, one-way ANOVA, **p < 0.01; ***p < 0.001, ****p < 0.0001. The phagocytosis activities of and *RNASET2*-silenced or parental THP-1 macrophages were tested by FACS analysis (*N* = 3), following FITC-dextran engulfment for 30, 60, 90, 120 min **(E).** Results at 120 min are shown as FACS histograms (**F**) and MFI dot-line graphs (**G**). As a control to block phagocytosis, both *RNASET2*-silenced or parental THP-1 macrophages were maintained at 4 °C, in presence of FITC dextran. Data are shown as mean ± SEM, student-T test, *p < 0.05

To further assess at the functional level the impact of RNASET2 on the macrophage cell lineage in the context of prostate cancer, we employed a co-culture approach (entailing a direct cell-to-cell contact) to mimic the RNASET2-mediated cancer cell/macrophage functional crosstalk occurring in vivo. For this purpose, we used THP-1-derived macrophages, whose endogenous levels of *RNASET2* expression were stably silenced by RNA interference (Supplementary Fig. 2D-E, as in [14]). *RNASET2* silencing did not affect THP-1 cell viability, that was comparable to that of parental THP-1 cells (Fig. 8A). 22RV1 and PC-3 cancer cells were thus grown in tightly contact with THP-1-derived macrophages.

To discriminate PCa cells to THP-1 macrophages in our co-culture system, pcDNA3-transfected 22Rv1 and PC-3 cells were co-transfected with an expression vector encoding GFP, were used, as target cells.

Our primary goal was to verify if control and *hRNASET2*-silenced THP-1 M0 macrophages differ in their antitumor activity (i.e. phagocytosis) on prostate cancer cells, in our co-culture system. We thus used parental and *RNASET2*-silenced THP-1 monocytes (the latter used either as a cellular pool or a single *hRNASET2*-silenced clone), following their differentiation into M0 macrophages. Then, 22RV1- and PC-3 GFP^+^ cells were added to the same culture well to establish direct cell-to-cell contact in co-cultures. We selected 2 clones for each prostate cancer cell line, namely clones 22Rv1/GFP^+^ #2 and #3, and clones PC-3/GFP^+^ #10 and #12. We then assessed the amount of live cancer cells, present on coverslips, after a 24-hours co-culture, by quantify the percentage of fluorescent area, due to cancer cell-derived GFP expression.

As shown in Fig. 8B-C (and quantified by ImageJ in Fig. 8D), a statistically significant increase in the fluorescent signal (representing viable PCa cells) was found for both 22Rv1 and PC-3 tested clones, when PCa cells were grown in presence of *hRNASET2*-silenced THP-1 M0 macrophages (for both cellular pool and clone #50), compared to PCa cells co-cultured with control, RNASET2 + THP-1 M0 macrophages (Fig. 8B, D).

To assess whether the reduced anti-tumor activities of *hRNASET2*-silenced THP-1 differentiated macrophages were related to impaired phagocytosis, we also tested the phagocytic activities of *hRNASET2*-silenced and parental THP-1 macrophages, within a 30- to 120-minute time frame (Fig. 8E-G). We observed that FITC-Dextran is less bound less at the surface of hRNASET*2*-silenced THP-1 differentiated macrophages, in line with the reduced FITC^+^ macrophages, as showed in Fig. 8E. This difference became statistically significant both in terms of % of FITC^+^ cells and MFI, at the 120-minute time point (Fig. 8E).

## Discussion

Prostate cancer (PCa) remains a major clinical challenge due to its molecular heterogeneity, the paucity of robust prognostic and predictive biomarkers, and the limited efficacy of existing therapies, particularly in advanced stages. Despite significant advances in androgen receptor (AR)-targeted treatments, therapeutic resistance, metastatic progression, and the immunologically “cold” nature of the TME continue to limit patient outcomes. Immunotherapeutic strategies have shown limited benefit in PCa as well, likely due to the immunosuppressive nature of the TME, where M2-like tumor-associated macrophages (TAMs) play a major role in supporting tumor progression and metastasis.

RNASET2 has emerged as a multifaceted tumor suppressor gene (TSG), known to exert both cell-autonomous and non-cell-autonomous effects in several cancer types [7, 9, 13, 24]. Following in-silico analysis, we found that high RNASET2 expression is associated with both improved progression-free interval (PFI) and a reduced risk of developing PCa. We also verified that RNASET2 expression is low across most available PCa cell lines. Accordingly, Kawahara et al. [40], using the updated Gleason scoring system, identified RNASET2 among eleven proteins differentially expressed across all five PCa grades and its expression was significantly downregulated in metastatic lesions compared to primary tumors.

Based on previous studies on the oncosuppressive activities of RNASET2 in different cancer types [7, 9, 13, 24], and deregulated expression of this gene in PCa, we investigated the potential cell autonomous and non-cell autonomous effects of RNASET2 in vitro and in vivo, using two well-establishedestablished PCa in vitro models, namely PC-3 and- 22Rv1 cells, selected base on their differential degrees of aggressiveness and clinical relevance, representing early-stage and advanced disease, respectively [41]. We observed that these two cancer cell lines showed a marked difference in their response to RNASET2 overexpression, likely modulated by intrinsic cellular features and tumor stage in PCa context.

As an initial assessment of potential cell-autonomous effects, we found that, RNASET2-overexpressing PC-3 cells demonstrated an increased proliferation rate, compared to empty controls, particularly at 48 h of growth, becoming less significant at 72 h, as assessed by crystal violet. To get deeper in our analyses, we moved to colony formation assay, that requires a longer time frame, more likely resembling the in vivo scenario. Using this assay, we observed no statistically significant difference between RNASET2-overexpressing PC-3 cells, compared to PC-3 empty cells. This suggest that RNASET2 can induce a transient increase in proliferation that, however, is loss in longer time frame. On the contrary, using the same approach, RNASET2-overexpressing 22Rv1 cells showed a robust and statistically significant reduced proliferation, maintained for all the 3 time points assessed, as detected by crystal violet assay. Corroborating these results, RNASET2-overexpressing 22Rv1 cells exhibited also a statistically significant reduction of generated colony, thus confirming the effects observed by crystal violet, but in a larger time frame. These findings suggest, for the first time in 22Rv1 cells, a functional link between RNASET2 and cell cycle regulation, confirmed by the statistically significant decrease of Cyclin-D1 protein levels in RNASET2-overexpressing 22Rv1 cells, compared to 22Rv1 empty cells.

We next investigated whether RNASET2 overexpression affected additional hallmarks of tumor progression, specifically cell adhesion and migration. In line with its anti-proliferative and anti-clonogenic effects, RNASET2-overexpressing 22Rv1 cells displayed significantly reduced adhesion compared to controls, although no differences in migration were observed. By contrast, RNASET2-overexpressing PC-3 cells showed enhanced adhesion and migration, reinforcing the divergent responses of the two cell models.

To further explore these differences, we assessed cytoskeletal remodeling, a key determinant of cell adhesion and motility [42, 43]. RNASET2-overexpressing PC-3 cells exhibited increased spreading coupled to the formation of migratory structures with lamellipodia- or filopodia-like appearance, consistent with their in vitro increased adhesion and migration rate and their metastatic phenotype *in vivo.* Conversely, RNASET2-overexpressing 22Rv1 cells showed the formation of cell clusters with peripheral accumulation of actin filaments, which might explain the observed decrease in their adhesion pattern. This finding aligns with previous studies in ovarian cancer models, where RNASET2 overexpression induced an ECM-related phenotype marked by reduced proliferation and distinct cytoskeletal organization [33]. The absence of this phenotype in PC-3 cells likely underlie their resistance to RNASET2-mediated tumor suppression. These observations suggest that differential expression of ECM-related features may contribute to the cell-type-specific effects of RNASET2 on cytoskeletal dynamics, adhesion, and migration in PCa.

To assess the impact of RNASET2 on pro-tumorigenic pathways, we analysed genes linked to EMT (CDH1, SNAIL, SLUG, TWIST, CTNNB1), metastasis (MMP9, YAP), and immune evasion (PD-L1). In 22Rv1 cells, RNASET2 overexpression led to the downregulation of all targets analysed, reaching statistical significance for TWIST, CTNNB1, MMP9, and YAP, supporting a broad regulatory role of RNASET2 in these cells. PC-3 cells displayed a similar trend of downregulation, for CDH1, SLUG, CTNNB1, and YAP although the effect was more limited since not reaching statistical significance.

We then explored potential non-cell-autonomous effects by profiling secreted factors. RNASET2-overexpressing 22Rv1 cells showed increased release of cytokines and chemokines involved in recruiting and activating immune cells, particularly monocytes and M1-polarized macrophages. A weaker effect was observed in PC-3 cells. These findings suggest that RNASET2 may contribute to shaping an anti-tumor immune microenvironment, although further validation is needed.

These results align with prior studies showing RNASET2’s context-dependent tumor-suppressive activity [7]. In this context, it is worth noting that, whereas the tumor suppressive activity of RNASET2 has been widely demonstrated both in vitro and in vivo across numerous cell lines representing a wide range of human cancer types, the few cell lines that turned out to be unresponsive to RNASET2-mediated suppression represented highly aggressive cancer subtype, in which most or all pathways involved in the highly pleiotropic oncosuppressive role of RNASET2 have likely been inactivated. This is possibly the main difference in the sensitivity to RNASET2 overexpression observed in our PCa cell lines.

The divergent responses of 22Rv1 and PC-3 cells to RNASET2 overexpression could reflect the molecular heterogeneity of PCa. While 22Rv1 cells, representing early-stage AR-positive PCa, show RNASET2 sensitivity, PC-3 cells, derived from aggressive, late-stage, AR-negative bone metastases, exhibit RNASET2 resistance [41]. Of note, RNASET2 is predominantly expressed in epithelial cells, aligning with 22Rv1 features. PC-3 cells lack PSA and AR, suggesting alternative oncogenic pathways drive their growth.

A key distinction between these cell lines is also represented by PTEN expression levels, since 22Rv1 cells express PTEN, while PC-3 cells are PTEN-deficient [44]. PTEN loss is linked to aggressive and drug-resistant PCa [45–47], thus RNASET2’s interaction with PTEN *(manca una referenza)* may underlie treatment response differences. The low response of PC-3 cells to RNASET2 could therefore stem from their intrinsic molecular characteristics, emphasizing the importance of selecting in vitro models that align with clinical disease features to better understand RNASET2’s role in PCa.

Consistent with our previous in vitro findings, RNASET2-overexpressing 22Rv1 cells displayed reduced tumorigenicity in nude mice. In contrast, no change in tumor growth was observed in mice injected with RNASET2-overexpressing PC-3 cells, confirming their resistance to RNASET2-mediated suppression. Importantly, tumors derived from RNASET2-overexpressing 22Rv1 cells showed an increased proportion of F4/80⁺CD80⁺ M1-like macrophages and a reduction in F4/80⁺CD206⁺ M2-like macrophages, resulting in a higher M1/M2 ratio. No such changes were detected in PC-3-derived tumors. These data are in keeping with the result of our secretome analysis and suggests that RNASET2 may promote a more immunostimulatory microenvironment in 22Rv1 tumors, likely linked to its ability to enhance the release of immunomodulatory soluble factors. In contrast, the microenvironment in PC-3 tumors appears less responsive to RNASET2-driven immune modulation.

These findings are consistent with our previous work in a syngeneic C51 colon carcinoma model, where murine RNASET2 overexpression delayed tumor growth and promoted recruitment of M1-like macrophages, suppression of M2-like macrophages and myeloid-derived suppressor cells (MDSCs), and expansion of CD8⁺ T cells [17]. This response was associated with long-term tumor rejection, underscoring RNASET2’s capacity to orchestrate effective antitumor immunity.

In particular, monocyte/macrophage lineage cells appear to play a central role in RNASET2’s activity. RNASET2 silencing in THP-1-derived human macrophages led to reduced antitumor activity against 22Rv1-but not PC-3-cells, likely due to impaired phagocytic function. This highlights a potentially conserved mechanism by which RNASET2 modulates the tumor microenvironment across different cancer models.

Taken together, our results reinforce the pleiotropic role of RNASET2 in mediating both cell-autonomous and immune-mediated tumor suppression.

## Conclusions

This study is to our knowledge the first to demonstrate the tumor-suppressive role of human RNASET2 in prostate cancer, acting through both cell-autonomous mechanisms and non-cell-autonomous pathways, particularly by promoting M1-like macrophage polarization. While our findings expand the spectrum of cancer types responsive to RNASET2, the lack of effect observed in PC-3 cells highlights the existence of RNASET2 resistance mechanisms, warranting further investigation. These results emphasize the critical influence of tumor-intrinsic factors on therapeutic responsiveness and underscore the need for precision medicine strategies that account for molecular heterogeneity within prostate cancer.

These findings support the potential of RNASET2 as a biomarker for patient stratification in prostate cancer, based on its expression and/or circulating levels in relation to tumor progression and immune responsiveness. Although our data are derived from overexpression models, they also suggest a possible translational application, where recombinant RNASET2 could be explored as a therapeutic agent to modulate the tumor microenvironment in selected PCa subsets.

## Electronic supplementary material

Below is the link to the electronic supplementary material.

**Supplementary Material 1**: **Supplementary table 1**: Sequences of the primers used for real-time PCR analysis. List and sequence of forward and revers human primers used for real-time PCR on PC-3 and 22Rv1 - empty and RNASET2-overexpressing - cells.

**Supplementary Material 2**: **Supplementary Table 2**: List of differential gene expressed in PCa tumor tissues (PC) Vs non-tumor tissues (HC) and relative *p*-value.

**Supplementary Material 3**: **Supplementary Fig. 1**: Basal RNASET2 expression in PCa cell lines. Graph showing RNASET2 expression in 8 PCa cell lines, as extracted from Protein Atlas (A); western blot confining the null expression of RNASET-2 in 4 different PCa cell lines and 1 BPH cell line, available in our laboratory.

**Supplementary Material 4**: **Supplementary Fig. 2**: Western blot for the detection of RNASET2 protein level in RNASET2-overexpressing and empty control PC-3 cells (**A**), and RNASET-overexpressing and empty vector control 22Rv1 cells (**B**) in cell pellets; western blot for the detection of RNASET2 protein level in RNASET-overexpressing and empty control PC-3 cells, and RNASET-overexpressing and empty control 22Rv1 cells in cell conditioned media (**C**). Western blot for the detection of RNASET2 protein level in THP-1 parental and RNASET-2 silenced cells (**D**), and related conditioned media (**E**).

**Supplementary Material 5**:** Supplementary Fig. 3**: C6 membrane array scan for RNASET-overexpressing and empty control PC-3 cells **(A**,** C**), and empty control and RNASET-overexpressing 22Rv1 cells **(B**,** D**); list of the targets spotted on the C6 membrane array (**E**).

## Data Availability

The raw data supporting the conclusions of this article will be made available by the corresponding author, without undue reservation.

## Abbreviations

CCL2: C-C motif ligand − 2
CM: Conditioned media
DC: Dendritic cell
FBS: Fetal bovine serum
GEO: Gene expression omnibus
HBV: Hepatitis B virus
HCV: Hepatitis C virus
MCP-1: Monocyte chemotactic protein
NK: Natural killer
PB: Peripheral blood
PCa: Prostate cancer
PMA: Phorbol 12-myristate 13-acetate
T reg: T regulatory
TAM: Tumor-associated macrophages
TME: Tumor microenvironment
