# Supplementary material for: The human RNASET2 alarmin-like molecule differentially affects prostate cancer cells behavior in both cell autonomous and non-cell autonomous manners
Source: J Transl Med. 2025 May 19;23:560. doi: 10.1186/s12967-025-06540-0 (PMC12090474; doi:10.1186/s12967-025-06540-0)
Supplement: Supplementary file 1 — Supplementary Material 1: Supplementary table 1: Sequences of the primers used for real-time PCR analysis. List and sequence of forward and revers human primers used for real-time PCR on PC-3 and 22Rv1 - empty and RNASET2-overexpressing - cells. [file 12967_2025_6540_MOESM1_ESM.docx]

**Supplementary Table 1**

| Primers target | Forward | Reverse |
| --- | --- | --- |
| *CDH1* | GCCTCCTGAAAAGAGAGTGGAAG | TGGCAGTGTCTCTCCAAATCCG |
| *SNAIL* | TGCCCTCAAGATGCACATCCGA | GGGACAGGAGAAGGGCTTCTC |
| *SLUG* | ATCTGCGGCAAGGCGTTTTCCA | GAGCCCTCAGATTTGACCTGTC |
| *TWIST* | GCCAGGTACATCGACTTCCTCT | TCCATCCTCCAGACCGAGAAGG |
| *CTNNB1* | CACAAGCAGAGTGCTGAAGGTG | GATTCCTGAGAGTCCAAAGACAG |
| *MMP-9* | GCCACTACTGTGCCTTTGAGTC | CCCTCAGAGAATCGCCAGTACT |
| *YAP* | TGCGTAGCCAGTTACCAACACTG | TCGAGAGTGATAGGTGCCACTG |
| *PD-L1* | TGCCGACTACAAGCGAATTACTG | CTGCTTGTCCAGATGACTTCGG |
| *18S* | GCAGAATCCACGCCAGTACAAG | GCTTGTTGTCCAGACCATTGGC |

**Supplementary Table 1. Sequences of the primers used for real-time PCR analysis.** List and sequence of forward and revers human primers used for real-time PCR on PC-3 and 22Rv1 - empty and RNASET2-overexpressing - cells.
